# The Dual Roles of Regulatory B Cells in Infection, Cancer, and Immunity

**DOI:** 10.1002/mco2.70850

**Published:** 2026-06-26

**Authors:** Anni Feng, Qilong Li, Ning Jiang, Qijun Chen

**Affiliations:** ^1^ Key Laboratory of Livestock Infectious Diseases，Ministry of Education College of Animal Science and Veterinary Medicine， Shenyang Agricultural University Research Unit for Pathogenic Mechanisms of Zoonotic Parasites of Chinese Academy of Medical Sciences Shenyang China

**Keywords:** chronic infections, IL‐10, regulatory B cells, tumor‐related immunity

## Abstract

Regulatory B cells (Bregs) are a functionally defined yet phenotypically heterogeneous subset of lymphocytes that are essential for maintaining immune homeostasis. Their canonical function is regulated through the secretion of interleukin‐10 (IL‐10), a potent anti‐inflammatory cytokine. However, accumulating evidence indicates that other molecules, such as IL‐35 and transforming growth factor‐β, and that of contact‐dependent pathways, such as Programmed Cell Death Ligand‐1 (PD‐L1) and Programmed Cell Death‐1 (PD‐1), also play indispensable roles in their regulatory arsenal. This review examines the immunoregulatory roles of Bregs across diverse clinical contexts, including infectious diseases, cancers, autoimmune disorders (such as systemic lupus erythematosus, rheumatoid arthritis, multiple sclerosis, and uveitis), and organ transplantation. Crucially, we highlight a fundamental functional dichotomy: although Bregs confer protection against autoimmunity and promote transplant tolerance, they concurrently drive the progression of chronic infections and malignancies by dampening antipathogen and antitumor immune responses. This functional dichotomy highlights the complexity of immune regulation, where Bregs act as critical nodes balancing health and pathology. The immense therapeutic potential by modulating Bregs activity and unresolved questions that will guide the future frontiers of Bregs research are essentially discussed.

## Graphical abstract

Regulatory B cells maintain immune homeostasis via suppressive cytokines and surface molecules like PD‐L1. While beneficially suppressing autoimmunity and promoting transplant tolerance, these identical mechanisms detrimentally attenuate antipathogen and antitumor immune responses. Consequently, this context‐dependent bimodal immunomodulatory profile establishes Bregs as critical therapeutic targets across distinct pathological states.

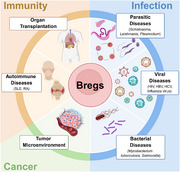

## 1 Introduction

B cells, the cornerstones of the adaptive immune system, are traditionally celebrated for their antibody‐producing capabilities [1, 2]. In recent years, however, their roles have expanded beyond that of mere humoral effectors to encompass multifaceted immunoregulatory functions. A subset known as regulatory B cells (Bregs) has emerged as a focal point of intense research because of their potent immunosuppressive capacity. This section will trace the evolution of the Breg concept, explore the central challenges in defining their identity, and provide an overview of their diverse inhibitory mechanisms, setting the stage for a deeper analysis of their complex roles in various physiological and pathological contexts.

The concept of Bregs originated in the 1970s when researchers observed that B cell‐depleted splenocytes were less effective in suppressing delayed‐type hypersensitivity reactions in guinea pigs upon adoptive transfer [3]. This seminal finding provided the first evidence that B cells possess a negative regulatory function independent of antibody production [4]. Although the underlying molecular mechanisms remained elusive at that time, these early studies laid the groundwork for the existence of “suppressive B cells.” This concept, however, did not gain significant traction for nearly three decades until the turn of the 21st century, when advances in molecular immunology allowed for the elucidation of its key mechanism. In a murine model of inflammatory bowel disease, the suppressive capacity of B cells was shown to be dependent primarily on the secretion of the anti‐inflammatory cytokine interleukin‐10 (IL‐10) [5]. This landmark discovery not only provided a solid molecular basis for “suppressive B cells” but also led to their rebranding as “regulatory B cells” to more accurately reflect their active roles in maintaining immune balance [5]. Concurrently, clinical observations provided indirect evidence for the presence of human Bregs. For instance, treatment with the B cell‐depleting agent rituximab in some patients with autoimmune disorders unexpectedly triggered or exacerbated other immune‐mediated conditions, such as psoriasis and ulcerative colitis [6, 7, 8]. This phenomenon suggested the presence of a protective B cell subset in humans, the absence of which could disrupt immune homeostasis and lead to uncontrolled inflammation. The journey of Bregs research—from functional observations to the elucidation of the molecular mechanism and clinical correlations—clearly illustrates how a scientific concept is progressively established and accepted through the mutual reinforcement of in vivo functional experiments, molecular identification, and clinical relevance. Unlike regulatory T cells (Tregs), which are defined by the specific lineage transcription factor Foxp3 [9, 10, 11], the Bregs field faces a central challenge: the lack of a single, stable molecular marker that uniquely identifies all Bregs [12]. The absence of a marker makes their definition, isolation, and tracking exceptionally complex and has sparked a profound debate about their fundamental nature: Are Bregs a distinct, terminally differentiated B cell lineage, or are they a functional state induced from various B cell precursors under specific microenvironmental cues?

Accumulating evidence supports the latter hypothesis that the term “Bregs” is now more widely considered a functional descriptor representing the anti‐inflammatory activity of B cells rather than a fixed cell lineage [13]. Studies have shown that Bregs can differentiate from multiple B cell developmental subsets, including transitional B cells from the bone marrow, mature peripheral B cells, splenic marginal zone B cells, and even activated plasmablasts [12]. This result suggests that the regulatory function of these cells is likely an inducible, dynamic state rather than a predetermined developmental endpoint. This diversity in origin directly leads to the findings of vast heterogeneity of Bregs phenotypes. In different disease models or physiological states, researchers have used various combinations of surface molecules to enrich for B cells with regulatory functions, such as the CD1d^hi^CD5^+^ combination, which is commonly used in autoimmune models, and the CD24^hi^CD38^hi^ or CD24^hi^CD27^+^ combination, which is used to identify Bregs precursors in human peripheral blood [14]. While this diverse phenotypic definition reflects the broad scope of Bregs function, it has also created confusion regarding the nomenclature, making cross‐study comparisons difficult. The “lineage vs. state” debate is not merely an academic exercise, it has profound implications for understanding Bregs biology and designing related therapeutic strategies. If Bregs were a distinct lineage, therapies would focus on specifically depleting or expanding this particular cell population. However, if Bregs represent a functional state, a more effective therapeutic approach might be to target the signaling pathways that induce or stabilize this regulatory state. For example, by modulating Toll‐like receptor (TLR) signaling, the cytokine environment, or tumor‐derived factors, one could functionally reshape the response propensity of B cells. This conceptual shift moves the therapeutic focus from the “cell” itself to the “signaling network” that governs its function.

Bregs employ a diverse set of molecular tools to execute their immunosuppressive functions, the complexity of which extends far beyond our initial understanding. IL‐10 is undoubtedly the cornerstone of the functional arsenal of Bregs and the most thoroughly studied effector molecule. As a potent anti‐inflammatory cytokine, IL‐10 can broadly inhibit the proinflammatory responses of various immune cells and is a central mediator of Bregs‐mediated immune homeostasis [13]. However, the regulatory capacity of Bregs is not solely dependent on IL‐10. Subsequent research has revealed a more intricate inhibitory network that includes other key immunosuppressive cytokines, such as IL‐35 and TGF‐β. IL‐35, a newer member of the IL‐12 family, can induce the expansion of both Tregs and B cells, resulting in the formation of a positive feedback inhibitory loop [15]. TGF‐β is a pleiotropic cytokine that, in specific contexts, can be produced by Bregs to effectively inhibit the differentiation of type 1 T helper (Th1) cells and type 17 Th (Th17) cells [16, 17]. Furthermore, Bregs can exert their effects through direct cell‐to‐cell contact. Programmed death‐ligand 1 (PD‐L1) expressed on Bregs can bind to programmed death 1 (PD‐1) on T cells, delivering an inhibitory signal [18, 19]. Fas ligand (FasL) expression can be induced in activated T cells [20]. These non‐IL‐10‐dependent mechanisms have greatly enriched our understanding of Bregs function, implying that Bregs can flexibly deploy different strategies to modulate the intensity and nature of immune responses according to the specific immune environment. These core mechanisms are detailed in the subsequent sections of this review.

This review traces the conceptual evolution of Bregs, emphasizing their phenotypic heterogeneity and the ongoing debate regarding whether they represent a distinct developmental lineage or an inducible functional state. We elucidate the primary mechanisms governing Bregs‐mediated immunosuppression and evaluate their dichotomous roles across infection, cancer, autoimmunity, and transplantation. Finally, we highlight emerging therapeutic strategies designed to target Bregs across diverse clinical contexts.

## 2 The Multifaceted Roles of Bregs

Bregs play multidimensional roles in the immune system, with their phenotype, function, and regulatory mechanisms exhibiting significant heterogeneity across different physiological and pathological conditions. This section will systematically dissect the complexity of Bregs, starting with their diverse subset characteristics and core inhibitory mechanisms, and then delving into their dual roles in key areas such as infectious diseases, tumor immunity, autoimmunity, and transplant tolerance.

### 2.1 Phenotypic and Functional Heterogeneity of Bregs Subsets

Due to the absence of a universal lineage marker, the identification of Bregs relies primarily on their function (e.g., IL‐10 production) and the expression of a combination of surface molecules. These phenotypic combinations vary across species and disease models, reflecting the intrinsic complexity of the Bregs population.

B10 cells are a typical subset of inhibitory B cells (Bregs), and their characteristics lies in the ability to rapidly produce IL‐10 in response to appropriate stimulation [21]. In mice, B10 cells are predominantly found within the splenic CD1d^hi^CD5^+^ B cell subset [22], which has been shown to play a critical inhibitory role in various autoimmune disease models, such as experimental autoimmune encephalomyelitis (EAE) [23]. In humans, the situation is different. B cells that directly produce IL‐10 are extremely rare in peripheral blood [24]. However, a population known as “B10 progenitor cells” (B10pro) can differentiate into functional B10 cells after 48 h of in vitro stimulation [25]. These B10pro cells are found mainly within the CD24^hi^CD27^+^ memory B cell pool [26]. This discovery suggests that human Bregs may exist more in a “latent” state, requiring specific inflammatory signals for B10 cell activation and function.

In addition to B10 cells, several other important Bregs subsets have been identified in mouse models. Transitional 2‐marginal zone precursor (T2‐MZP) Bregs with a CD19^+^CD21^hi^CD23^hi^CD24^hi^ phenotype are considered a significant source of Bregs [27]. In some disease models such as collagen‐induced arthritis (CIA), T2‐MZP Bregs suppress disease progression by producing IL‐10, indicating that B cells can acquire regulatory potential at early developmental stages [28].

T cell immunoglobulin and mucin domain‐containing protein 1 (TIM‐1) is a crucial immunoregulatory molecule [29, 30]. Studies have shown that a substantial portion of IL‐10‐producing B cells in mice express TIM‐1 and its expression is not limited to a specific B cell subset but is widely distributed among transitional, marginal zone, and follicular B cells [31]. Activation of TIM‐1 signaling can induce Bregs expansion and promote immune tolerance, making it a potential therapeutic target [31].

In humans, research has also revealed multiple B cell subsets with regulatory functions, the most prominent of which include CD19^+^CD24^hi^CD38^hi^ transitional B cells and CD19^+^CD24^hi^CD27^+^ and memory B cells. The subset of CD19^+^CD24^hi^CD38^hi^ transitional B cells is composed mainly of immature B cells that have recently exited the bone marrow. In healthy individuals, these cells are capable of producing IL‐10 and suppressing Th1/Th17 responses [32]. However, in autoimmune diseases such as systemic lupus erythematosus (SLE) and type 1 diabetes [33, 34, 35], this subset is numerically or functionally deficient and cannot effectively suppress autoreactive T cells, which is considered a key mechanism contributing to autoimmunity. As mentioned earlier, CD19^+^CD24^hi^CD27^+^ memory B cells consist of the progenitor cells of human B10 cells and are a major source of IL‐10 [36]. They play a crucial role in maintaining peripheral immune tolerance. As research progresses, more Bregs subsets with specialized functions are being discovered. For example, a subset known as Br1 cells (phenotype CD25^hi^CD71^hi^CD73^lo^) mediates immune tolerance in allergic reactions by producing IL‐10 and allergen‐specific IgG4 antibodies [37]. In the tumor microenvironment (TME), a subset of Bregs expressing Granzyme B (GrB) has been shown to suppress antitumor T cell activity through the secretion of GrB [38].

Bregs represent an inducible functional state characterized by potent immunosuppressive activity in both mice and humans, rather than a distinct developmental lineage with a unifying phenotypic signature. Under inflammatory conditions, B cells at diverse developmental stages can acquire immunoregulatory capabilities. These functions are mediated predominantly via IL‐10 secretion, alongside the deployment of other effector molecules, including IL‐35, TGF‐β, Tim‐1, TIGIT, and FasL, to modulate T cells, dendritic cells, and other immune populations. Although these core functional principles are conserved, substantial biological and methodological disparities distinguish murine and human Bregs research. Canonical murine Bregs subsets are relatively well defined, permitting the direct establishment of causality through in vivo disease models, genetic manipulation, and adoptive transfer paradigms. Conversely, human Bregs exhibit profound heterogeneity, extreme context‐dependency, and an absence of universal specific markers. Consequently, human studies remain heavily reliant on peripheral blood analyses and ex vivo induction assays, which structurally limits the depth of definitive mechanistic insight. We systematically summarize the major Bregs identified in mice and humans, detailing their phenotypes, effector molecules, and associated disease contexts (Tables 1 and 2).

**TABLE 1:** Phenotypes and functions of major regulatory B cell subsets in mice.

Subset name	Key phenotypic markers	Major effector molecules	Primary research context/model
B10 Cells	CD1d^hi^CD5^+^	IL‐10	EAE [23, 39], contact hypersensitivity [40]
T2‐MZP Bregs	CD19^+^CD21^hi^CD23^hi^CD24^hi^(IgM^hi^IgD^+^CD1d^+^)	IL‐10	Collagen‐induced arthritis (CIA) [28]
Plasmablasts	CD19^+^CD27^int^CD38^+^	IL−10	EAE [41]
Killer B cells	CD19^+^CD5^hi^FasL^hi^		HCV [42]
MZ Bregs	CD19^+^CD21^hi^CD23^−^	IL‐10	Parasites [43]
TIM‐1^+^ Bregs	TIM‐1^+^	IL‐10	Transplant tolerance [31], EAE [44], parasites [45]
CD5^+^CD1d^+^	CD19^+^CD5^+^CD1d^hi^	IL‐10	Parasites [46, 47]
i35‐Bregs	Undefined	IL‐35	EAE [48], EAU [49]
i27‐Breg	CD23^−^CD5^+^p28^+^EBI3^+^	IL‐27	EAE, EAU [50]

Abbreviations: EAE, experimental autoimmune encephalomyelitis; HCV, hepatitis C virus; EAU, experimental autoimmune uveitis.

This table clearly illustrates the current state of the Bregs field: on the one hand, IL‐10, a core effector molecule, is common to multiple Bregs subsets, demonstrating its functional universality; on the other hand, different subsets show significant differences in phenotype, other effector molecules (e.g., IL‐35 and FasL), and associated pathological contexts. This heterogeneity is not chaotic but suggests that the immune system recruits and activates B cell subsets with specific functions to perform “tailor‐made” regulatory tasks in response to different challenges.

**TABLE 2:** Phenotypes and functions of major regulatory B cell subsets in human.

Subset name	Key phenotypic markers	Major effector molecules	Primary research context/model
B10/memory Bregs	CD19^+^CD24^hi^CD27^+^	IL‐10	Healthy individuals, autoimmune diseases [26], CHB [51]
Transitional Bregs	CD19^+^CD24^hi^CD38^hi^	IL‐10, TGF‐β	SLE [33], type 1 diabetes [52], HIV [53], CHB [54], and non‐infectious uveitis [55]
Br1 cells	CD25^hi^CD71^hi^CD73^lo^	IL‐10, IgG4	Allergic diseases [37]
GrB^+^ Bregs	CD19^+^CD38^+^CD1d^+^IgM^+^	Granzyme B, IL‐10	Solid tumors [38]
Regulatory memory B cell	Tim‐1^+^TIGIT^+^		MS [56]
CD5^+^CD1d^+^	CD19^+^CD5^+^CD1d^hi^	IL‐10	Bacteria [57]
i35‐Bregs	Undefined	IL‐35	SLE [58]
i27‐Breg	CD20^+^CD27^+^CD43^+^CD11b^+^	IL‐27	Uveitis, neuroinflammation [50]

Abbreviations: CHB, chronic hepatitis B; SLE, systemic lupus erythematosus; HIV, human immunodeficiency virus; MS, multiple sclerosis.

Major human Bregs subsets are classified according to their phenotypic markers, major effector molecules, and primary research contexts or disease models. Distinct Breg subsets mediate immunoregulatory functions mainly through the production of IL‐10, TGF‐β, IL‐27, IL‐35, and granzyme B.

### 2.2 Core Mechanisms of Bregs‐Mediated Suppression

Bregs exert their immunosuppressive functions through a diverse and coordinated set of molecular mechanisms. These processes can be broadly categorized into two types: long‐range regulation via soluble cytokines and short‐range, contact‐dependent inhibition via cell surface molecules.

#### 2.2.1 Cytokine‐Dependent Regulation

IL‐10 is a hallmark cytokine produced by Tregs and exerts a wide range of anti‐inflammatory effects [59]. First, it directly acts on helper T cells, effectively inhibits the differentiation and function of proinflammatory Th1 and Th17 cells and thereby reduces the production of cytokines such as IFN‐γ and IL‐17 [60]. IL‐10 can also suppress the activation of myeloid cells [61] such as monocytes [62], macrophages, and dendritic cells [63] and downregulate the expression of MHC Class II [64] and costimulatory molecules, impairing their antigen‐presenting capacity and reducing the secretion of proinflammatory factors such as TNF‐α. Furthermore, IL‐10 is a key factor inducing the differentiation of Tregs and type 1 regulatory T cells (Tr1) [65]. By secreting IL‐10, Bregs can expand these T cell subsets, which also have inhibitory functions, thereby amplifying the immunosuppressive effect [66, 67].

The ability of Bregs to produce IL‐10 is precisely regulated by multiple signals [4], with B cell receptor (BCR)‐mediated antigen recognition, TLR (especially TLR4 and TLR9) sensing of pathogen‐associated molecular patterns, and CD40–CD40L interaction with T cells being the three core signaling pathways that induce IL‐10 expression [41, 68, 69]. This signal integration mechanism ensures that Bregs activation is synchronized with the initiation of the immune response, forming a classic negative feedback loop: the signals that drive inflammation also simultaneously activate the mechanisms that suppress it, thus preventing an excessive immune reaction.

IL‐35 is a heterodimeric cytokine composed of Epstein–Barr virus‐induced gene 3 (EBI3) and p35 subunits and belongs to the IL‐12 family with potent immunosuppressive activity [70]. Initially IL‐35 was thought to be exclusively produced by Tregs, subsequent studies revealed that B cells are also a significant source of IL‐35, and these cells are termed i35‐Bregs [71]. IL‐35 exerts a uniquely “infectious” effect. It not only suppresses the function of effector T cells but also induces the conversion of naive T and B cells into IL‐35‐producing regulatory cells, thereby creating a self‐amplifying inhibitory network in the local inflammatory environment [72]. In B cells, IL‐35 signals through its receptor (composed of IL‐12Rβ2 and IL‐27Rα) to activate the signal transducer and activator of transcription 1 (STAT1) and STAT3 pathways, which in turn promotes the conversion of B cells to an IL‐35‐ and IL‐10‐producing regulatory phenotype [73].

IL‐27 serves as a critical effector molecule in Bregs‐mediated immunoregulation. As a heterodimeric member of the IL‐12 cytokine family, IL‐27 is composed of the p28 and EBI3 subunits. Crucially, the EBI3 subunit is shared with IL‐35, another IL‐12 family member; however, despite this structural homology, these two cytokines orchestrate distinct biological functions [74]. Recent studies underscore the potent immunosuppressive capacity of IL‐27 across diverse autoimmune pathologies. In experimental models of EAE and experimental autoimmune uveitis (EAU), IL‐27 ameliorates disease severity by concomitantly dampening pathogenic Th1 and Th17 responses—alongside downregulating GM‐CSF production—whilst driving the differentiation of IL‐10‐producing Tr1 cells [75, 76, 77]. Recently, a distinct subset of IL‐27‐producing Bregs (i27‐Bregs), originated from the B‐1a compartment, has been identified. Beyond constitutively secreting IL‐27, these cells amplify their immunosuppressive capacity by upregulating coinhibitory receptors, including PD‐1 and LAG3. Functionally, i27‐Bregs not only dampen pathogenic Th1 and Th17 inflammatory responses but also orchestrate the conversion of conventional B cells into IL‐10‐ or IL‐35‐producing regulatory phenotypes. Through these coordinated mechanisms, i27‐Bregs confer robust protection against disease pathogenesis in experimental models of EAE and EAU [50, 78]. In summary, IL‐27 plays a crucial role in limiting inflammatory responses and maintaining immune homeostasis by restricting effector T cell‐mediated inflammation on one hand, while enhancing Tregs function on the other, and serving as an upstream inducer of IL‐10 to promote its production.

TGF‐β is a pleiotropic cytokine with complex effects on B cells; it can inhibit B cell proliferation and class switching of most antibodies while promoting IgA production [79]. As an effector molecule of Bregs, B cell‐derived TGF‐β plays a key inhibitory role in certain disease models. For example, in an EAE model, Bregs‐produced TGF‐β indirectly suppressed Th1 and Th17 autoimmune responses by reducing the antigen‐presenting capacity of dendritic cells [80]. Additionally, TGF‐β is important for inducing Treg cell differentiation and functions in concert with IL‐10 to maintain immune tolerance [81, 82].

#### 2.2.2 Contact‐Dependent Inhibition and Apoptosis Induction

Programmed cell death protein 1 (PD‐1) and its ligand PD‐L1 are well‐known immune checkpoints crucial for maintaining T cell functional homeostasis [83, 84]. Bregs can express high levels of PD‐L1 [19, 85, 86]. When they act as antigen‐presenting cells and interact with PD‐1‐expressing activated T cells, PD‐L1/PD‐1 engagement delivers a strong inhibitory signal to T cells, leading to T cell exhaustion or anergy [19]. This mechanism is particularly important in chronic viral infections (such as human immunodeficiency virus [HIV]) and the TME, where Bregs use this pathway to help viruses or tumors evade T cell clearance [87, 88].

A subset of Bregs (sometimes called “killer B cells”) can express FasLs (FasL and CD178) [89]. Fas (CD95) is a death receptor expressed on the surface of activated T cells. When FasL^+^ Bregs recognize and present a specific antigen to the corresponding T cells through their BCR, the binding of FasL to Fas on the T cells can directly initiate the apoptosis of T cells [90]. This mechanism efficiently and irreversibly eliminates autoreactive or overly activated effector T cells. The specific receptor–ligand interaction also ensures the precision of the suppressive action, avoiding damage to unrelated immune cells [91].

#### 2.2.3 Other Inhibitory Functions of Bregs

GrB is a serine protease that is traditionally known as a primary weapon of cytotoxic T cells and NK cells for killing target cells [92]. However, research has shown that, particularly in the TME, a subset of Bregs can also secrete GrB [38]. Unlike cytotoxic lymphocytes, GrB secreted by Bregs may not directly lyse target cells but degrade the ζ‐chain of the T‐cell receptor (TCR), thereby impairing the TCR signaling capacity and ultimately inducing T‐cell apoptosis [38].

Bregs can express two key ectonucleotidases on their surface: CD39 and CD73 [93]. CD39 hydrolyzes proinflammatory extracellular ATP into AMP, and subsequently, CD73 hydrolyzes AMP into adenosine. Adenosine is a potent immunosuppressive molecule that can inhibit T cell proliferation and cytokine production by acting on adenosine receptors on the T cell surface [94]. Through this pathway, Bregs can convert a “danger signal” (ATP) in the inflammatory microenvironment into a “suppressive signal” (adenosine).

These diverse inhibitory mechanisms form a complex regulatory network, enabling Bregs to adopt the most appropriate strategy for different immune challenges. Cytokine‐mediated mechanisms are suitable for broad, paracrine‐style suppression in inflammatory regions; contact‐dependent mechanisms allow for precise, “point‐to‐point” regulation of T cells on an antigen‐specific basis, and mechanisms that directly induce apoptosis represent the ultimate means of eliminating harmful effector cells. This functional specialization indicates that Bregs are not single‐function cells but rather a multifunctional “toolbox” deployed by the immune system to maintain homeostasis.

### 2.3 Bregs in the Crucible of Infection: A Double‐Edged Sword

In the process of fighting against pathogens, the core task of the immune system is to effectively eliminate the invaders while minimizing damage to its own tissues. As the “brakes” of the immune responses, the role of Bregs becomes particularly complex and contradictory in this context. They can either protect the host by suppressing excessive inflammation or be exploited by pathogens to facilitate immune evasion and chronic colonization.

#### 2.3.1 Bregs in Parasitic Diseases

Chronic parasitic infections, especially helminth infections, are classic examples of host–pathogen coevolution characterized by complicated maneuver in host–parasite interaction [95]. Bregs play a central role in this process.

**FIGURE 1:**
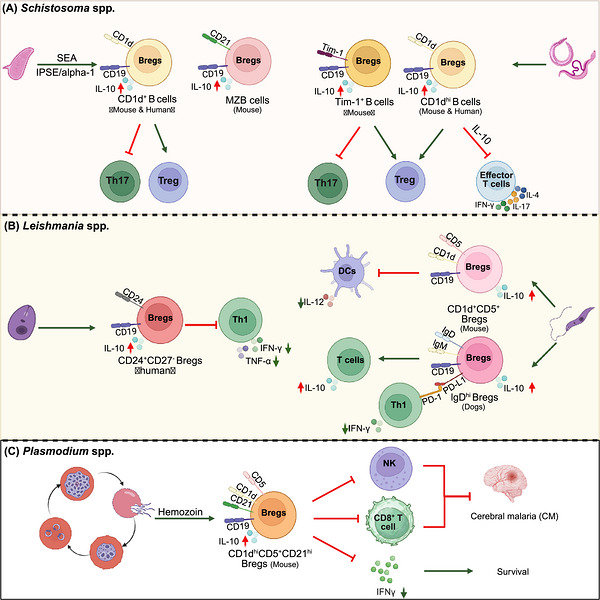
The immunomodulatory role of Bregs in infections caused by *Schistosoma*, *Leishmania*, and *Plasmodium* parasites. (A) Schistosome eggs promote the secretion of IL‐10 by CD1d^+^ B cells and marginal zone B (MZB) cells through the schistosome egg antigen IPSE/alpha‐1. CD1d^+^ B cells facilitate the differentiation of Tregs by secreting IL‐10 while also suppressing Th17 differentiation. Adult worms induce the production of CD1d^hi^ B cells and Tim‐1^+^ B cells, leading to increased IL‐10 secretion, which enhances immune tolerance by promoting Treg development. (B) The amastigote stage of *Leishmania* stimulates human B cells to differentiate into CD24^+^CD27^−^ Bregs and secrete IL‐10 while concurrently inhibiting the production of proinflammatory cytokines such as IFN‐γ and TNF‐α by Th1 cells. The promastigote stage of *Leishmania* promotes the secretion of IL‐10 from CD1d^+^CD5^+^ Bregs and LgD^hi^ Bregs, inhibits the secretion of IL‐12 by dendritic cells, and suppresses the production of IFN‐γ by Th1 cells, thereby inducing T cells to secrete IL‐10. (C) The hemozoin of *Plasmodium* stimulates B cells to differentiate into CD1d^hi^CD5^+^CD21^hi^ Bregs, promoting their survival by inhibiting natural killer (NK) and CD8^+^ T cells while reducing IFN‐γ levels.

In schistosomiasis, schistosome egg antigen (SEA) is among the most potent known inducers of Bregs (Figure 1A). During infection, SEA continuously stimulates B cells to differentiate into IL‐10‐producing Bregs [96, 97, 98]. These Bregs significantly reduce pathological damage from granulomas formed around the eggs by suppressing Th17 inflammatory responses, thereby protecting vital organs such as the liver [98, 99, 100, 101]. However, this immune tolerance comes at the cost of ineffective clearance of adult worms, allowing the parasite to persist in the host [97, 102]. Recent studies have shown that schistosomes can also promote the generation of Tregs, induce IL‐10 expression by Tim‐1^+^B cells and suppress IL‐17 secretion by T cells [45].

Control of *Leishmania* infection depends on the host's ability to mount an effective Th1‐type cellular immune response. However, *Leishmania* infection also induces the expansion of Bregs (Figure 1B), particularly IL‐10‐producing subsets [103, 104]. These Bregs potently inhibit Th1 cell differentiation and function, thereby weakening the ability of dendritic cells to kill parasites and leading to chronic infection and lesion progression [103, 105]. In animal models, the number of Bregs is positively correlated with disease susceptibility, and depleting Bregs or blocking IL‐10 signaling significantly enhances host resistance [40].

Malaria is an acute, highly inflammatory parasitic disease [106]. During *Plasmodium* infection, Bregs expand significantly (Figure 1C), and their numbers correlate with the level of parasitemia in the peripheral blood [107]. On the one hand, these Bregs may contribute to parasite proliferation and evasion by suppressing antimalarial T cell immunity. On the other hand, malaria infection is often accompanied by a severe cytokine storm and immunopathology. In some animal models, adoptive transfer of Bregs, while potentially temporarily increasing the parasite load, can significantly reduce mortality by suppressing excessive inflammation [108]. This finding perfectly illustrates the dual role of Bregs in acute infections, by impairing pathogen clearance but also containment of immunopathology.

#### 2.3.2 Bregs in Viral Diseases

**FIGURE 2:**
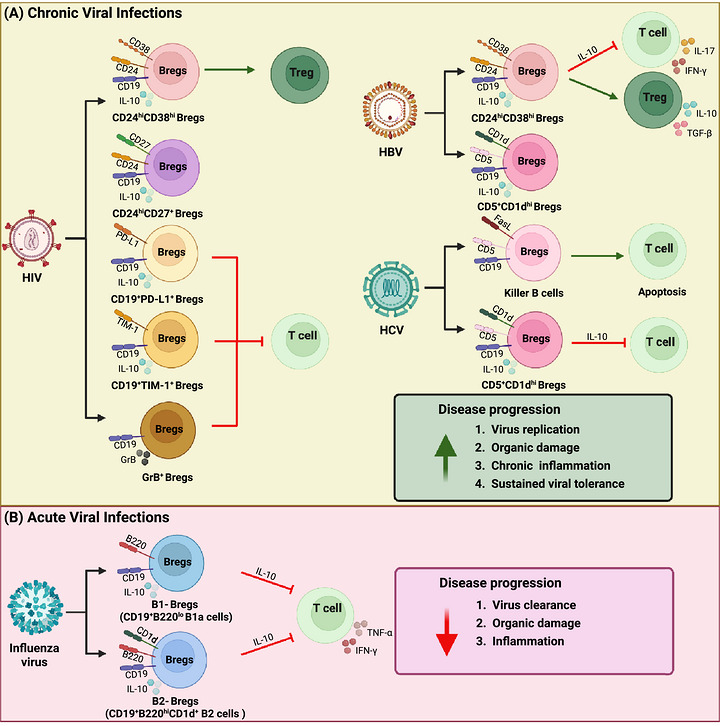
Distinct immunomodulatory functions of Bregs during chronic or acute viral infections. (A) During chronic viral infections, such as HIV, HBV, and HCV infections, Bregs contribute to the maintenance of immune balance by promoting the Treg‐mediated T cell suppression through the secretion of IL‐10. Bregs alleviate inflammation while simultaneously facilitating viral replication, thereby inducing persistent viral tolerance. (B) In acute viral infections such as influenza, Bregs secrete IL‐10 to inhibit the secretion of proinflammatory cytokines by T cells, including TNF‐α and IFN‐γ, which mitigates pulmonary immunopathology.

The role of Bregs in viral infections largely depends on whether the infection is acute or chronic. In chronic infections caused by HIV [109], hepatitis B virus (HBV) [110], and hepatitis C virus (HCV) [111, 112], the expansion of Bregs is generally associated with a poor prognosis (Figure 2A).

In early HIV infection, the number of IL‐10‐producing Bregs increases and is positively correlated with the viral load. HIV infection induces a chronic elevation of serum B cell‐activating factor (BAFF) that remains unabated despite suppressive antiretroviral therapy (ART). This persistent BAFF excess disrupts B‐cell developmental homeostasis, specifically attenuating the regulatory potential of MZP‐like B cells [113]. Concurrently, it downregulates the expression of critical immunoregulatory molecules, including IL‐10, CD83, and members of the NR4A family, thereby compromising overall Breg functionality. Furthermore, the sustained dysregulation of the BAFF/APRIL axis during prolonged ART propagates systemic chronic inflammation and drives non‐AIDS‐defining comorbidities, notably subclinical atherosclerosis [114]. In parallel, HIV‐infected individuals exhibit a disrupted Breg‐to‐Treg balance characterized by profound functional heterogeneity within the Bregs compartment. Although their baseline capacity to suppress CD4^+^ T cell proliferation is markedly impaired [109], there is a paradoxical expansion of the CD19^+^CD24^hi^CD38^hi^ Breg subset, which positively correlates with viremia, systemic immune activation, and CD8^+^ T cell exhaustion. Mechanistically, this expanded subset leverages IL‐10 and PD‐L1 to comprehensively suppress antigen‐presenting cells, bulk CD4^+^ T cells, and HIV‐specific CD8+ T cell responses, ultimately facilitating viral persistence and exacerbating immune exhaustion [115]. GrB^+^Bregs [116] and TIM‐1^+^Bregs [117] also contribute to immune regulation in HIV, similarly suppressing T‐cell responses to mediate immune inhibition. These Bregs are likely hijacked by the virus for establishment of persistent infection and immune evasion by effectively suppressing HIV‐specific T cell responses [87].

In chronic HBV infection, peripheral Bregs are markedly expanded, particularly in patients during the immune‐active phase [118, 119, 120]. These cells are typically defined as IL‐10‐producing B cell subsets, with the most commonly reported phenotype being CD19^+^CD24^hi^CD38^hi^ [51, 110], while other phenotypes, such as CD19^+^CD5^+^CD1d^hi^ B cells, are also elevated in patients with chronic HBV, and also in HCV infections [111]. Functionally, Bregs directly suppress HBV‐specific CD8^+^ T cell responses, which are essential for the clearance of infected hepatocytes, primarily through IL‐10‐mediated inhibition of IFN‐γ and IL‐17 production, thereby attenuating antiviral effector function [121]. Beyond IL‐10, Bregs can induce T cell dysfunction via IL‐35, contributing to immune suppression and imbalance during chronic infection [122]. Meanwhile, Bregs induce the differentiation of CD4^+^CD25^−^ T cells into Tregs and upregulate the expression of CTLA‐4, IL‐10, and TGF‐β, suggesting their collaboration with Tregs in constructing an immunosuppressive network. Bregs may also indirectly affect humoral immune responses by regulating follicular helper T cells (Tfh) [123].

Similarly, in chronic HCV infections, the number of Bregs is significantly increased in both peripheral blood and liver tissue and is closely related to the degree of liver fibrosis and disease progression [42, 51]. By producing inhibitory cytokines such as IL‐10, they promote T cell exhaustion and immune tolerance [124, 125, 126].

Unlike chronic infections, one of the main threats to the hosts during acute influenza virus infection is the excessive inflammatory response, or “cytokine storm,” which can lead to severe lung immunopathology and even death [127]. In this scenario, the immunosuppressive function of Bregs may be beneficial. Although research on the role of Bregs in influenza infection is still limited, Bregs could theoretically mitigate lung tissue damage by producing IL‐10 to suppress excessive inflammatory cell infiltration and cytokine production in the lungs, thus striking a balance between viral clearance and tissue protection (Figure 2B) [128, 129]. The role of B cells in immune responses to influenza is multifaceted, from early antigen transport to later antibody production, all of which are crucial [130].

#### 2.3.3 Bregs in Bacterial Diseases

In bacterial infections, the function of Bregs also mediate a trade‐off between controlling the pathogen and limiting inflammatory damage. The early phase of bacterial sepsis is characterized by an uncontrolled systemic inflammatory response. In animal models of bacterial sepsis, Bregs, particularly innate‐like B‐1a cells, have been shown to exert a significant protective effect by producing large amounts of IL‐10 to counteract hyperinflammatory reaction [131]. In septic mice, deficient in B‐1a cells or Bregs have higher mortality rates [132], whereas the reinfusion of B‐1a cells improves survival [131, 133]. This finding indicates that in acute, systemic bacterial infections, the “braking” function of Bregs is crucial for host survival.

**FIGURE 3:**
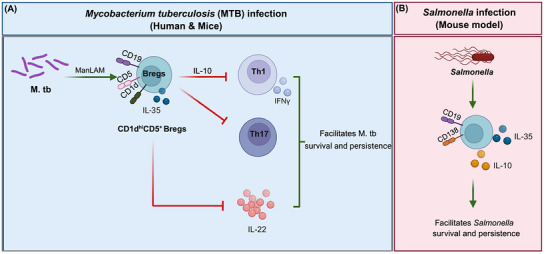
The immunomodulatory role of Bregs in *Mycobacterium tuberculosis* and *Salmonella* infections. (A) *M. tuberculosis* can induce the differentiation of Bregs through its ManLAM. Bregs suppress the production of IFN‐γ by Th1 cells through the secretion of anti‐inflammatory cytokine IL‐10, while also inhibiting Th17 cells and IL‐22 secretion, thereby weakening the host's antibacterial immune response. These effects collectively facilitate the survival and persistent infection of *M. tuberculosis* within the host. Similarly, during *Salmonella* infection, CD19^+^CD138^+^ Bregs can be induced. These cells exert immunosuppressive effects by secreting IL‐10 and IL‐35, creating favorable conditions for the survival and persistent colonization of the bacteria.

The pathological hallmark of *Mycobacterium tuberculosis* infection is the formation of granulomas, structures composed of immune cells designed to contain bacteria locally [134]. B cells are important components of these granulomas [135]. Recent studies suggest that B cells within granulomas do not directly kill bacteria but instead indirectly regulate the bactericidal function of macrophages through the recruitment and organization of T cells (particularly Tfh‐like cells) [136]. The major surface glycolipid of *M. tuberculosis*, mannose‐capped lipoarabinomannan (ManLAM), potently drives IL‐10 production by Bregs. This ManLAM‐induced Breg activation subsequently blunts Th1 cell polarization, thereby facilitating mycobacterial evasion and long‐term persistence within the host [137]. In tuberculosis patients, CD19^+^CD1d^+^CD5^+^ Bregs suppress T‐cell responses beneficial for antituberculosis immunity [138]. Standard antituberculosis treatment can partially restore IL‐22‐related immune functions by reducing these B cells [57]. Moreover, in patients with active tuberculosis, B cells exhibit a stronger capacity of IL‐35 production [139], and CD5^+^ Bregs (CD19^+^CD24^hi^CD38^hi^CD5^+^) are significantly reduced [140]. The role of Bregs in this context is not fully understood, but researchers have speculated that they may prevent tissue necrosis and damage by suppressing excessive inflammation within the granuloma while also potentially weakening the ability to clear bacteria (Figure 3A).

*Salmonella* is a group of intestinal pathogens that can induce strong CD4^+^ T‐cell‐mediated immune responses [141]. However, *Salmonella* has also evolved mechanisms to evade B‐cell‐mediated control [142]. B cell‐derived IL‐10 regulates immune responses during *Salmonella* infection through the MyD88 signaling pathway, exerting immunosuppressive effects that prevent effective bacterial clearance [143]. These findings establish IL‐10 as a core mediator of immunosuppression and immune evasion during *Salmonella* infection. Mechanistically, IL‐10‐ and IL‐35‐producing Bregs actively subvert host clearance pathways to establish a persistent carrier state [48], a critical pathological adaptation that facilitates ongoing pathogen transmission (Figure 3B).

In summary, the role of Bregs in infectious diseases reveals an intense battle between the host and pathogen centered on immune regulation. Bregs are components of a key homeostatic mechanism evolved by the host to prevent self‐injury, but many pathogens, especially those that tend to establish chronic infections, have evolved strategies to specifically and potently induce Bregs. They hijack the host's self‐protective mechanism to promote pathogen survival. Furthermore, the dynamics of infections (acute vs. chronic) are critical variables in determining whether Bregs function is beneficial or detrimental. In acute infections where immunopathology is the primary threat, the inhibitory function of Bregs is beneficial, whereas in chronic infections where pathogen persistence is the main problem, the same inhibitory function becomes harmful.

### 2.4 The Protumorigenic Function of Bregs

In the field of tumor immunology, a growing consensus is that Bregs play key roles in tumor immune evasion and disease progression. They actively suppress antitumor immunity through multiple mechanisms, thus acting as “traitors” in the TME.

#### 2.4.1 Infiltration and Function in the TME

A solid tumor mainly composed of cancer cells is a complex ecosystem in the microenvironment, which includes many immune cells, stromal cells, and blood vessels, among other components [144]. Bregs can be recruited to the TME by chemokines secreted by the tumor cells or can be converted from conventional B cells within the TME through the “education” of tumor‐associated signaling [145]. Once in the TME, Bregs become a major force in the construction of an immunosuppressive microenvironment. In many human cancers, including lung, breast, ovarian, and pancreatic cancer, the extent of Bregs infiltration in tumor tissue is often correlated with late‐stage disease, a high risk of metastasis, and a poor prognosis for patients [145].

#### 2.4.2 Bregs and Antitumor Immunity

Bregs have been found to negatively deploy their comprehensive “arsenal” to disrupt the antitumor immune response in the TME [146]. Bregs potently suppress tumor‐infiltrating CD8^+^ cytotoxic T lymphocytes [147] and CD4^+^ Th1 cells [148, 149]. They directly inhibit the proliferation, activation, and cytokine production [150] (e.g., IFN‐γ and TNF‐α) of these effector T cells by secreting the signature inhibitory cytokines IL‐10, TGF‐β, and IL‐35 [151, 152]. Additionally, Bregs utilize contact‐dependent mechanisms, such as inducing T cell exhaustion through the binding of overexpressed PD‐L1 to PD‐1 on T cells [153] or directly inducing T‐cell apoptosis through the secretion of GrB to degrade the T cell receptor [38].

Bregs do not act alone but form a complex inhibitory network with other immunosuppressive cells in the TME, such as Tregs [154, 155], myeloid‐derived suppressor cells [156], and tumor‐associated macrophages [157]. The mutual promotion between Bregs and Tregs is particularly critical. Bregs can efficiently induce the differentiation of naive CD4^+^ T cells into Foxp3^+^ Treg cells and promote the expansion and functional maintenance of existing Tregs through the production of IL‐10 and TGF‐β [158]. The collaboration between these two major regulatory lymphocyte populations significantly increases the overall immunosuppressive strength of the TME, thereby creating an environment conducive to tumor growth.

Tumor development is a dynamic process of “immunoediting,” comprising three phases: elimination, equilibrium, and escape. Bregs play a particularly prominent role in this process, and tumors rely on these cells during the “escape” phase. In the early stages of tumorigenesis, effector B cells may participate in antitumor immunity by presenting tumor antigens to other cytotoxic immune cells or secreting antitumor antibodies. However, under continuous immune pressure, surviving tumor cells evolve multiple immunosuppressive mechanisms, including the active secretion of specific factors to “educate” or induce infiltrating B cells to transform into Bregs. These “tumor‐educated” Bregs, in turn, suppress T cells that could have eliminated the tumor, thereby helping the tumor ultimately escape immune control.

**FIGURE 4:**
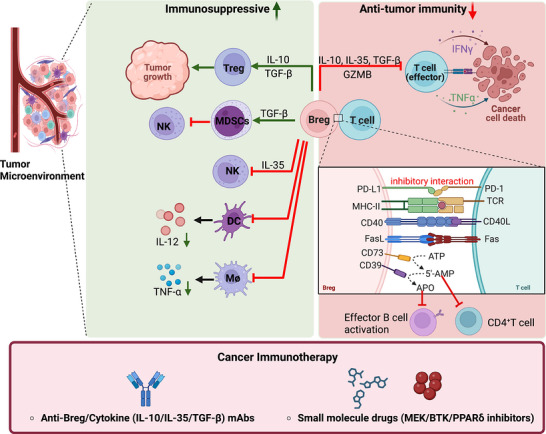
Bregs play a role in promoting tumor development. In the tumor microenvironment, Bregs contribute to tumor development by enhancing immunosuppression and decreasing antitumor immunity. They secrete IL‐10 and TGF‐β to promote the expansion of Tregs, as well as TGF‐β and IL‐35 to inhibit NK cells. Additionally, Bregs suppress the secretion of the cytokines IL‐12 and TGF‐α from dendritic cells (DCs) and macrophages (Mø) while also inhibiting effector T cells. This multifaceted approach effectively prevents T cell‐mediated antitumor immunity, thereby facilitating tumor progression.

Given their central role in tumor immunosuppression, Bregs have become a highly attractive, new target for cancer immunotherapy (Figure 4). Current mainstream immune checkpoint inhibitors (e.g., anti‐PD‐1/PD‐L1 antibodies) [86, 159] primarily aim to “release” suppressed T cells [160]. However, many patients do not respond to or develop resistance to such therapies. The presence of Bregs provides a plausible explanation for this primary or acquired resistance, as they can suppress T cells through multiple non‐PD‐1‐dependent, redundant pathways (e.g., IL‐10, TGF‐β or Treg induction). Therefore, the development of strategies targeting Bregs, such as monoclonal antibodies that specifically deplete Bregs or small‐molecule drugs that inhibit Bregs function, and their combination with existing T cell checkpoint inhibitors are expected to produce synergistic effects, overcome resistance, and bring hope to more patients with cancer [161].

### 2.5 Bregs as Guardians of Immune Homeostasis

In stark contrast to their detrimental roles in chronic infections and cancers, Bregs play an indispensable protective role in maintaining self‐tolerance, preventing autoimmune diseases, and promoting transplant tolerance. In these contexts, Bregs are the “guardians” in the immune system, and their functional impairment or absence is a key factor leading to immune imbalance and pathological damage.

#### 2.5.1 Dysregulation and Functional Deficits in Autoimmune Diseases

The fundamental characteristic of autoimmune diseases is the attack on self‐tissues by the immune system, which usually stems from a breakdown of immune tolerance [162]. In contrast to the hyperfunctional state of Bregs in chronic infections and cancers, the core pathological mechanism of many autoimmune diseases is either reduction in Bregs numbers or functional deficiency [163].

**FIGURE 5:**
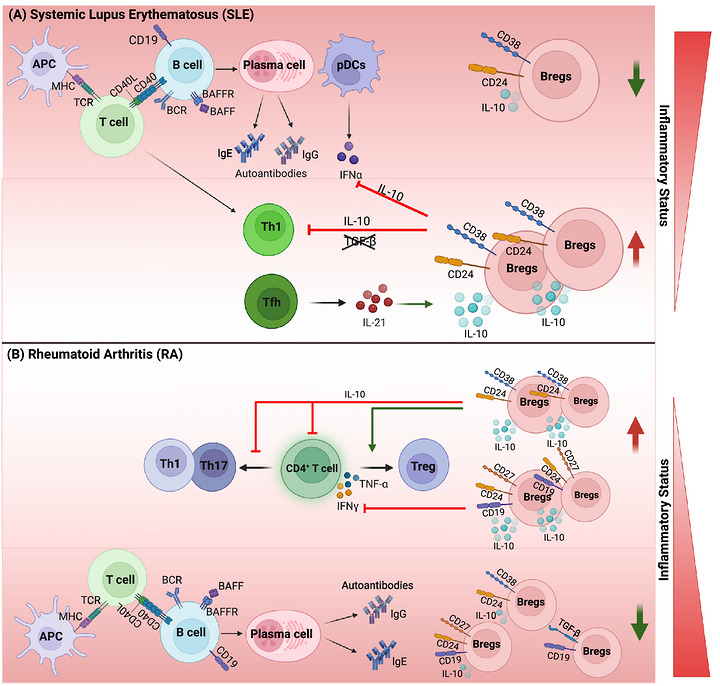
Bregs in systemic lupus erythematosus (SLE) and rheumatoid arthritis (RA). During the progression of SLE (A) and RA (B), a notable decrease in the number of Bregs occurs in response to increasing inflammation. An increase in the number of Bregs may mitigate disease progression by suppressing T cells or plasmacytoid dendritic cells (pDCs) and their associated proinflammatory cytokines through the secretion of IL‐10 or by promoting the expansion of Tregs.

SLE is a type of autoimmune disease characterized by the production of autoantibodies and multiorgan damage [164]. B cells play a central pathogenic role in SLE and differentiate into plasma cells that produce autoantibodies [165, 166]. However, not all B cells are harmful. In patients with lupus nephritis (LN), the number of Bregs is significantly reduced [167] and closely correlated with disease severity [168]. Meanwhile, Bregs in SLE patients often exhibit functional defects, such as decreased IL‐10 production capacity [169]. In mouse models of SLE with atherosclerosis, the reduction of Bregs is also associated with an imbalance in the Th17/Treg ratio [170]. Studies have shown that in patients with active SLE, the number of Breg subsets in the peripheral blood (such as CD24^hi^CD38^hi^ transitional B cells [171]) is significantly reduced, or their ability to produce IL‐10 is impaired (Figure 5A), rendering them unable to effectively suppress the activation of autoreactive T cells and the production of proinflammatory cytokines [172]. This loss of regulatory function is considered a key link in the breakdown of self‐tolerance and the sustained amplification of autoimmune responses.

Rheumatoid arthritis (RA) is an autoimmune disease primarily characterized by chronic inflammation of the synovial joints [173]. Like in patients with SLE, defects in Bregs are also observed in patients with RA (Figure 5B) [174]. A meta‐analysis of multiple studies confirmed that compared with healthy controls, the proportions of specific Bregs subsets (such as CD19^+^TGFβ^+^[175], CD19^+^CD24^hi^CD38^hi^ [176, 177], and CD19^+^CD24^hi^CD27^+^ B cells [178, 179]) in the peripheral blood of RA patients are significantly decreased [180]. Not only are their numbers reduced, but the Bregs in these patients also have impaired functions, as their ability to suppress T cell proliferation and IFN‐γ secretion is significantly weaker than that of Bregs from healthy individuals [32, 181]. This deficiency in Bregs function undoubtedly contributes to chronic inflammation and destruction of the joint tissues.

Multiple sclerosis (MS) is a chronic autoimmune pathology driven by neuroinflammation, progressive demyelination, and neuronal degradation within the central nervous system [182]. In individuals with MS, the immunoregulatory capacity of TIM‐1‐ and TIGIT‐expressing memory Bregs is profoundly impaired in peripheral circulation, highlighting their critical role in maintaining systemic immune homeostasis [56, 183]. Insights from EAE, the canonical murine model of MS [184], reveal that Bregs orchestrate tolerance primarily via the secretion of IL‐10 [163], IL‐35 [49], and IL‐27 [50]. These cytokines concurrently attenuate pathogenic Th1 and Th17 responses while driving Treg expansion [185], although IL‐10‐producing Bregs can also exert robust immunosuppression via Treg‐independent mechanisms [39]. Furthermore, Breg suppressive capacity is highly plastic and can be augmented by specific endogenous cues, notably GM‐CSF and IL‐15 [186]. Consequently, targeted modulation of Breg function, achieved via pharmacological interventions or autologous cell therapies, represents a promising frontier for MS immunotherapy.

Uveitis encompasses a spectrum of severe intraocular inflammatory conditions that are predominantly autoimmune in etiology [187]. During acute disease flares, patients exhibit both a numerical depletion and functional exhaustion of peripheral Bregs, notably the CD19^+^CD24^hi^CD38^hi^ subset, underscoring their indispensable role in enforcing ocular immune privilege and constraining deleterious inflammation [55]. The anti‐inflammatory efficacy of Bregs in this context is critically dependent on STAT3‐mediated signaling, which drives the sustained secretion of IL‐10 and IL‐35 [188]. Corroborating clinical observations, mechanistic studies utilizing the EAU model demonstrate that distinct Breg subsets—including CD1d^hi^CD5^hi^ IL‐10‐producing B cells [189] and i35‐Bregs [190]—potently resolve intraocular inflammation. These specialized populations achieve disease amelioration by coordinately suppressing inflammatory leukocyte activation, blunting effector T cell responses, and facilitating the regional expansion of complementary regulatory networks.

Notably, the functional deficits of Bregs in autoimmune diseases may not be an intrinsic genetic issue but rather a consequence of the chronic inflammatory environment. In SLE and RA patients, the persistent presence of proinflammatory cytokines (such as IFN‐γ, BAFF, and IL‐21) not only drives the differentiation of pathogenic B cells (such as autoantibody‐secreting cells) but also may actively inhibit the differentiation of B cells toward a regulatory phenotype or directly impair the function of existing Bregs [174, 191]. These findings suggest that therapeutic strategies designed to restore Bregs function may need to be combined with therapies that suppress the underlying inflammation to achieve the optimal results.

#### 2.5.2 Bregs‐Mediated Immune Regulation in Transplant Tolerance

**FIGURE 6:**
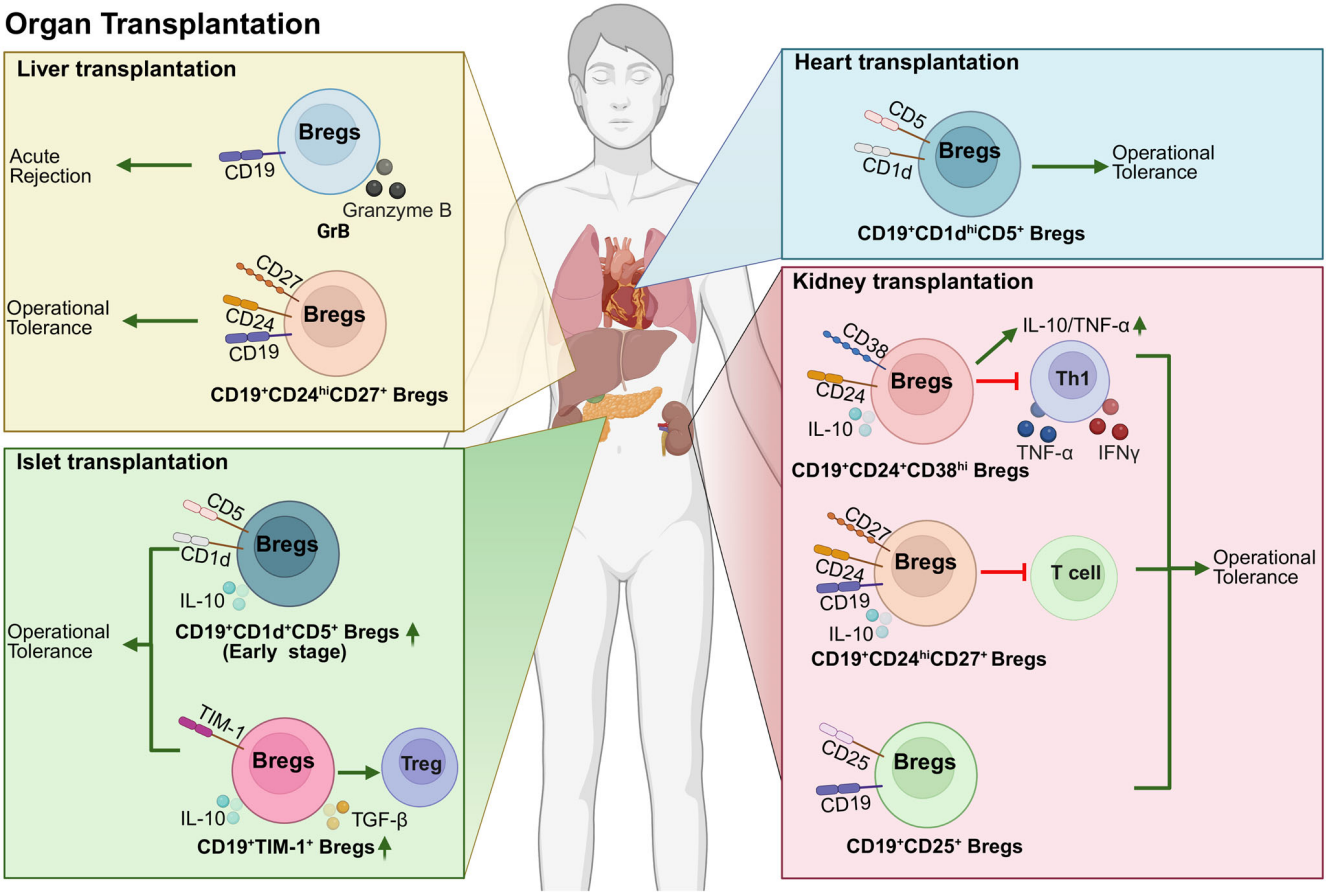
Bregs promote immune tolerance in patients with liver, heart, islet, and kidney transplantation. After transplantation, GrB‐producing B cells correlate with acute rejection of the transplant, whereas distinct Breg subsets—including CD19^+^CD24^hi^CD27^+^ Bregs in liver transplants, CD19^+^CD1d^hi^CD5^+^ Bregs in heart transplants, CD19^+^CD1d^+^CD5^+^ or TIM‐1^+^ Bregs in islet transplants, and CD19^+^CD24^+^CD38^hi^, CD24^hi^CD27^+^, or CD25^+^ Bregs in kidney transplants—promote immune tolerance in different organ grafts.

Maintenance of immune tolerance in the recipient toward the donor graft will eliminate the need of long‐term reliance on broad‐spectrum, toxic immunosuppressive drugs. In this context, Bregs have shown immense therapeutic potential (Figure 6).

Clinically, a small number of transplant patients were found to be able to maintain long term and stable graft function after a short usage of immunosuppressants, a state known as “operational tolerance” [192, 193]. Immunological studies of these patients have revealed that their peripheral B cells exhibit a unique “tolerant” signature that is enriched in CD24^hi^CD38^hi^ transitional B cells [194, 195], and the ability of these B cells to produce IL‐10 upon stimulation is significantly greater than that observed in typical transplant patients or healthy individuals [196, 197, 198]. These findings indicate that functionally robust Bregs may be critical for establishing and maintaining transplant tolerance.

In numerous animal transplant models, the protective role of Bregs has been more directly demonstrated. Adoptive transfer of Bregs can significantly prolong the survival of allografts (such as those of the heart and liver) and even induce long‐term tolerance [199, 200, 201]. Bregs employ their classic inhibitory mechanisms, such as secreting IL‐10 and TGF‐β, to suppress the differentiation of effector T cells that attack the graft and to promote the generation of protective Treg cells [202].

The role of Bregs in autoimmune reaction and transplant tolerance mirrors their roles in infection and cancer. This elegant symmetry eloquently demonstrates that Bregs are a core “pressure valve” of the immune system. In cancer or chronic infections, the immune system fails because Bregs‐mediated suppression is “too strong”; in autoimmune diseases, the failure is due to Bregs‐mediated suppression being “too weak,” and in organ transplantation, the therapeutic goal is to artificially create a state of “too strong” suppression of alloantigens. The final outcome of these diseases depends on which end of the spectrum this pressure valve is set.

## 3 Discussion and Future Perspectives

After more than two decades of rapid development, the study of Bregs has evolved from a relatively niche area to a central theme in immunology. We now recognize that Bregs are a functionally potent but phenotypically complex population with profound dual roles in health and disease. This section will synthesize the preceding content to distill the intrinsic logic of Bregs function, identify the unresolved questions facing researchers in this field, and provide perspectives on the future therapeutic applications.

### 3.1 Synthesizing the Duality of Bregs: Context Determines Function

The central paradox of Bregs function—being a protective “friend” in some situations and a pathogenic “foe” in others—can be unified by a core principle: context determines function. The role of a Breg cell is not solely determined by its phenotype but is dynamically shaped by the specific immunological environment it inhabits. Key contextual factors include the nature of the antigen, which can be classified as self, foreign, or tumor; the duration of stimulation, categorized as acute or chronic; and the local cytokine milieu. By secreting the same molecule (e.g., IL‐10), the same type of Bregs can exert a therapeutic effect on the joints of a RA patient by suppressing autoreactive T cells that attack the cartilage. However, in the melanoma microenvironment, these cells promote disease progression by suppressing the antitumor T cells that can kill cancer cells [148]. This functional shift is rooted in the different core challenges faced by the immune system in different scenarios. In autoimmune diseases, the challenge is excessive, self‐directed inflammation; in cancer, the immune response is insufficient. As an intrinsic brake of the immune system, the benefit or foe of Bregs action depends entirely on whether the timing and intensity of the “brake” match the current needs.

For a more intuitively summary of the roles of Bregs in different diseases and their therapeutic implications, the following Table 3 encapsulates the core content of this review.

**TABLE 3:** Summary of the roles of Bregs in major pathological states.

Pathological state	Primary role of Bregs	Key suppressive factors	Main therapeutic implication	References
Cancer	Pathogenic	IL‐10, TGF‐β, PD‐L1, Treg induction	Inhibit or deplete Bregs	[203, 204, 205]
Chronic viral infections (HIV/HBV)	Pathogenic	IL‐10, PD‐L1, T cell exhaustion	Inhibit or deplete Bregs	[117, 119]
Systemic lupus erythematosus (SLE)	Protective (functionally deficient)	IL‐10, TGF‐β	Expand Bregs or restore their function	[33, 191]
Rheumatoid arthritis (RA)	Protective (functionally deficient)	IL‐10, TGF‐β	Expand Bregs or restore their function	[206]
Organ transplantation	Protective	IL‐10, Treg induction	Expand Bregs or use for adoptive therapy	[207, 208]
Sepsis (acute phase)	Protective	IL‐10	Enhance Bregs function	[209, 210]

This table clearly reveals the translational logic of Bregs research: in diseases where Bregs play a “pathogenic” role, therapeutic strategies should aim to functional suppression, whereas in diseases where Bregs play a “protective” role, strategies should aim to functional expansion.

### 3.2 Unresolved Questions in Bregs Biology

Despite significant progress, many fundamental questions in the Bregs field remain unanswered, and their resolution will be the focus of future research. (1) Is there a dedicated B cell precursor committed to the Bregs lineage? or is the Bregs phenotype a reversible functional state that most B cells can enter when exposed to specific signals? What are the key transcription factors and epigenetic modifications that regulate Bregs fate decisions? (2) Once a B cell acquires regulatory function, is this state a stable endpoint, or can it revert to an effector phenotype in response to environmental changes? Can functionally deficient Bregs in autoimmune diseases be “re‐educated” into potently suppressive Bregs in the TME? (3) What are the similarities and differences in the functions of Bregs in different tissue microenvironments (such as the gut, skin, central nervous system, and solid tumors)? Are there tissue‐resident Bregs subsets with unique functions? And (4) How does the gut microbiota, one of the largest immunomodulatory factors in the body, influence the development, localization, and function of mucosal and systemic Bregs [27, 211, 212]?

### 3.3 Therapeutic Targeting on Bregs

A deeper understanding of Bregs function has opened new avenues for the treatment of several major diseases. Targeting Bregs is a promising strategy for enhancing antitumor or antipathogen immunity [213]. Although no absolutely specific surface markers for Bregs are currently available, depleting antibodies could be designed to target molecules enriched on Bregs (such as TIM‐1 and CD5). A more feasible approach might be to use small‐molecule inhibitors to block signaling pathways crucial for Bregs induction and function, such as TLR [214], STAT3 [201, 215], or CD40 signaling pathways [216, 217]. Combining these Bregs inhibitory strategies with existing immune checkpoint inhibitors is expected to overcome resistance and achieve the “dual liberation” of T cells.

For diseases with insufficient Bregs function, the therapeutic goal is to “supplement” Bregs. This process can be achieved through adoptive cell therapy, in which a patient's own B cells are induced and expanded into functional Bregs cells in vitro and then reinfused into the patient [218]. Researchers at the University of Oxford have developed a clinically applicable method to mass‐produce human Bregs and have demonstrated plausible efficacy of these cells in prolonging graft survival in humanized mouse models. Additionally, pharmacological agents, such as specific TLR agonists, recombinant cytokines (e.g., IL‐35), or drugs that selectively promote Bregs differentiation, can be used to enhance the function of endogenous Bregs in vivo [49, 219].

### 3.4 Challenges and Strategies in the Clinical Application of Bregs

Although Bregs hold significant therapeutic potential in autoimmune diseases, organ transplantation, and cancer treatment, their clinical application still faces numerous challenges. These obstacles primarily stem from the inherent biological complexity of Bregs, the lack of standardized isolation, purification, and expansion methods, as well as their context‐dependent (disease‐modulated) functional characteristics. Addressing these issues is crucial for translating Bregs‐based therapies from laboratory practice to clinical application.

#### 3.4.1 Major Challenges

A primary obstacle in the clinical application of Bregs is the absence of specific and stable surface markers. Unlike Tregs, which are defined by Foxp3 expression, Bregs lack a unified, lineage‐specific master transcription factor or canonical surface signature. Currently, Bregs are delineated by complex combinations of surface molecules (such as CD19^+^CD24ʰ^i^CD38ʰ^i^ or CD19^+^CD24ʰ^i^CD27^+^) that fluctuate significantly across disparate subsets, tissue microenvironments, and pathological states. This profound phenotypic heterogeneity fundamentally impedes the reproducible isolation of highly pure Breg populations for adoptive cell therapy and severely complicates the precise monitoring of in vivo Breg dynamics.

Compounding this issue is the inherent functional instability of these cells. Emerging paradigms dictate that the Breg phenotype does not represent a terminal lineage commitment, but rather a highly plastic, inducible, and reversible functional state. Governed by prevailing inflammatory or tumor‐derived microenvironmental cues, conventional B cells can acquire immunoregulatory properties; conversely, established Bregs can undergo phenotypic conversion, losing their suppressive capacity or transdifferentiating into proinflammatory effector cells upon exposure to specific cytokine milieus. This extreme plasticity raises critical concerns regarding the long‐term stability, efficacy, and safety of adoptively transferred Bregs in clinical settings.

Furthermore, as delineated in preceding sections, Bregs exhibit profound context‐dependency, exerting diametrically opposing effects based on the underlying pathology. Although they confer protection in autoimmune and allograft settings, they actively drive pathogenesis during chronic infections and malignancies. This functional dichotomy dictates that therapeutic interventions must be exquisitely tailored to the specific disease etiology and stage. Broad, non‐specific immunomodulatory strategies risk catastrophic disease exacerbation; for example, the untargeted expansion of Bregs in an oncological context could inadvertently precipitate tumor immune evasion and accelerate metastatic progression.

Finally, the therapeutic manipulation of Bregs presents substantial safety liabilities. The sustained immunosuppressive capacity of these cells inherently elevates the risk of secondary infections or de novo malignancies. In the context of transplantation or autoimmunity, prolonged Breg expansion could critically compromise host protective immunity, predisposing patients to opportunistic pathogens or the reactivation of latent viruses. Conversely, systemic Breg depletion strategies employed in cancer immunotherapy could precipitate severe autoimmune sequelae or unbridled inflammatory syndromes. These profound risks underscore the urgent necessity for rigorous safety monitoring protocols and the engineering of precisely controllable therapeutic modalities to safeguard patient outcomes.

#### 3.4.2 Potential Strategies to Overcome These Challenges

To address the phenotypic ambiguity of Bregs, the identification of lineage‐specific markers via comprehensive multi‐omics profiling is imperative. Advanced single‐cell technologies, including single‐cell RNA sequencing, mass cytometry, and high‐resolution epigenomic mapping, offer unprecedented opportunities to define stable Breg‐specific signatures. The integration of multi‐omics datasets may elucidate novel surface molecules—such as the combinatorial expression of TIM‐1, TIGIT, or CD73—that reliably discriminate Bregs from other B cell subsets across diverse pathophysiological contexts. The establishment of these markers would critically enable the precise isolation, in vivo tracking, and therapeutic targeting of these cells. Furthermore, to circumvent the inherent functional plasticity of Bregs, genetic engineering strategies, including the CRISPR‐mediated knock‐in of IL‐10 or IL‐35 under the control of constitutive or inducible promoters, can be employed to generate phenotypically locked Breg products with sustained suppressive capacity. Concurrently, the integration of synthetic biology principles, notably the incorporation of inducible suicide genes (such as iCasp9) into Breg products, provides a crucial fail‐safe mechanism to ablate transferred cells in the event of adverse reactions, thereby ensuring clinical safety.

Given their functional dichotomy, Breg‐based therapeutics must be exquisitely tailored to the specific pathological context, precluding the viability of a uniform clinical approach. In the settings of autoimmunity and allograft transplantation, therapeutic strategies must aim to augment both Breg frequency and suppressive function. This can be achieved via the adoptive transfer of autologous or allogeneic Bregs, or alternatively, through targeted in vivo expansion utilizing low‐dose IL‐35, rapamycin, or highly specific TLR modulators. Conversely, in the context of malignancies and chronic infections, the selective depletion of Bregs utilizing antibody–drug conjugates directed against Breg‐enriched surface targets presents a rational therapeutic avenue. Furthermore, the pharmacological blockade of critical differentiation pathways, such as STAT3 or specific TLR signaling cascades, may yield substantial clinical benefit. Crucially, integrating targeted Breg modulation with established immune checkpoint blockade regimens holds immense translational promise for circumventing current mechanisms of therapeutic resistance.

### 3.5 Conclusions

Bregs, key regulatory nodes in the immune system, have greatly expanded our understanding of B cell biology and the mechanisms of immune tolerance. From being initially overlooked as “suppressive cells” to now being recognized as a core force in maintaining immune balance, the story of Bregs is a microcosm of the development of modern immunology. The profound functional duality they exhibit in different diseases not only reveals the subtlety and complexity of immune regulation but also provides us with unprecedented therapeutic opportunities. The future challenge lies in moving beyond a simple “good” versus “bad” classification of Bregs to establish a more refined and dynamic framework that depends on specific spatiotemporal contexts. Elucidating the molecular switches that govern the transition of B cells between effector and regulatory fates will be the key to developing the next generation of precision immunotherapies and conquering a range of major human diseases, from autoimmunity to cancers.

## Author Contributions

*Writing – original draft and visualization*: Anni Feng. *Conceptualization and writing – original draft*: Qilong Li. *Writing – review and editing*: Ning Jiang. *Conceptualization, writing – review and editing, visualization, and funding acquisition*: Qijun Chen. All authors have read and approved the final version of the manuscript.

## Funding Information

This work was supported by the National Nature and Science Foundation of China (82530079) and The Research Unit for Pathogenic Mechanisms of Zoonotic Parasites, Chinese Academy of Medical Sciences, Shenyang, China (2019‐I2M‐5‐042).

## Ethics Statement

The authors have nothing to report.

## Conflicts of Interest

The authors declare no conflicts of interest.

## Data Availability

No datasets were generated or analyzed during the current study.
